# Smoking Trends among U.S. Latinos, 1998–2013: The Impact of Immigrant Arrival Cohort

**DOI:** 10.3390/ijerph14030255

**Published:** 2017-03-02

**Authors:** Georgiana Bostean, Annie Ro, Nancy L. Fleischer

**Affiliations:** 1 Sociology Department and Environmental Science & Policy Program, Chapman University, Orange, CA 92866, USA; 2 Program in Public Health, University of California, Irvine, CA 92697, USA; 3 Center for Social Epidemiology and Population Health, Department of Epidemiology, School of Public Health, University of Michigan, Ann Arbor, MI 48109, USA

**Keywords:** smoking, migration, Hispanic paradox, acculturation, cohort effects

## Abstract

Few studies examine nativity disparities in smoking in the U.S., thus a major gap remains in understanding whether immigrant Latinos’ smoking prevalence is stable, converging, or diverging, compared with U.S.-born Latinos. This study aimed to disentangle the roles of period changes, duration of U.S. residence, and immigrant arrival cohort in explaining the gap in smoking prevalence between foreign-born and U.S.-born Latinos. Using repeated cross-sectional data spanning 1998–2013 (U.S. National Health Interview Survey), regressions predicted current smoking among foreign-born and U.S.-born Latino men and women (n = 12,492). We contrasted findings from conventional regression analyses that simply include period and duration of residence effects, to two methods of assessing arrival cohort effects: the first accounted for baseline differences in smoking among arrival cohorts, while the second examined smoking probabilities by tracking foreign-born arrival cohorts as they increase their duration of U.S. residence. Findings showed that Latino immigrants maintained lower prevalence of current smoking compared with U.S.-born Latinos over the period 1998–2013, and that longer duration of U.S. residence is associated with lower odds of smoking among men. Two findings are particularly novel: (1) accounting for immigrant arrival cohort dampens the overall protective effect of duration of residence among men; and (2) the earliest arrival cohort of Latino immigrant men experienced the steepest decline in smoking over duration of U.S. residence. Results have methodological and theoretical implications for smoking studies and the Latino mortality paradox.

## 1. Introduction

Latino immigrants have lower mortality rates compared with U.S.-born Latinos and Whites [1], even though they have lower socioeconomic profiles on average. This advantage appears to be driven by fewer smoking-attributable deaths among foreign-born Latinos compared with U.S.-born Whites, suggesting that smoking plays a key role in maintaining the so-called “Latino mortality paradox” [2]. The few studies that have examined nativity have consistently documented lower smoking prevalence among foreign-born individuals living in the U.S. compared with U.S.-born Latinos and Whites [3,4,5,6]. However, a major gap remains in understanding whether immigrant Latinos’ smoking prevalence is stable, converging, or diverging, from that of U.S.-born Latinos. Understanding this issue will provide crucial insight into whether the Latino mortality advantage will continue to persist into the future. To fully understand this issue, we must consider three factors affecting smoking patterns: period trends, duration of U.S. residence, and immigrant arrival cohort effects.

Examining period trends in smoking among immigrants relative to the U.S.-born is important because smoking prevalence has declined substantially since the 1990s in the U.S. and many Latin American countries, as a result of strengthening tobacco control policies. These changes in tobacco policy, such as increased tobacco taxes, could contribute to the secular decline in smoking, in addition to the decline that often occurs with aging. However, it is not known whether the decline in smoking prevalence has been of similar magnitude for foreign-born Latinos as for U.S.-born Latinos. If smoking is declining faster among immigrants than the U.S.-born, this would suggest a widening nativity gap in smoking over time, with immigrants’ advantage increasing. On the other hand, if smoking is declining faster among the U.S.-born than the foreign-born, this would lead to the nativity gap decreasing, which may erode immigrants’ smoking and mortality advantage in the future.

However, immigrants’ smoking trends are shaped by additional time-related factors that are not applicable to the U.S.-born. For immigrants, the passage of time additionally reflects increasing duration of U.S. residence, which has generally been tied to worsening health behaviors over time [7]. However, smoking may be an exception to this general pattern of unhealthy assimilation, and the effect of duration of residence on smoking may vary by gender. Studies have found higher smoking prevalence with increased duration of residence (i.e., an unhealthy assimilation effect) among Latina women [7], but either no association or a slight decrease in smoking prevalence with increasing duration of residence among Latino men [8,9].

What is more, duration of residence effects among immigrants are primarily studied using cross-sectional data, which conflates the effect of time in the U.S. with immigrant arrival cohort effects—that is, differences between immigrants who arrived in the U.S. at different points in time [10]. For example, if a study using 2010 data found differences in smoking between immigrant groups with five years and 20 years U.S. residence, this may not be due to immigrants’ behaviors changing over increasing duration of residence, but rather to compositional differences between immigrants who entered the United States in 2005 versus 1990. Prior studies have found important differences between immigrant arrival cohorts in health outcomes including body mass index [11] and self-rated health [12]. However, few studies have examined this with respect to smoking. This is a particularly relevant source of bias considering the trend of declining smoking in U.S. Latino immigrants’ origin countries. For example, smoking has declined substantially in Mexico and Puerto Rico (two major immigrant sending countries) since the 2000s [13,14]. If recent immigrants reflect the smoking decline of their sending countries, more recently arrived immigrant cohorts should have lower smoking prevalence than prior cohorts. The differences in smoking prevalence may also translate into unique duration patterns for individual arrival cohorts. For instance, if a cohort arrives with high smoking prevalence, the decline in smoking may be steeper than for a cohort arriving with already low smoking prevalence.

Thus, our understanding of immigrant smoking trends relative to the U.S.-born is complicated by the fact that these multiple sources of variation in immigrants’ smoking prevalence—period, duration of residence, and cohort effects—are often unaccounted for in studies using conventional cross-sectional approaches. To address this issue, the present study aims to disentangle the roles of period changes, duration of U.S. residence, and immigrant arrival cohort in explaining the gap in smoking prevalence between foreign-born and U.S.-born Latinos. Our findings will contribute to existing literature by illuminating whether the nativity difference in smoking has changed over time (as smoking has declined in the U.S. and Latin American countries), how smoking prevalence differs between U.S.-born Latinos and foreign-born Latinos by duration of U.S. residence, and how immigrants’ duration of U.S. residence and immigrant arrival cohort influence the nativity gap in smoking.

Specifically, we use a novel approach by combining repeated cross-sectional data spanning 1998–2013 to examine smoking trajectories among foreign-born and U.S.-born Latinos. This approach is a middle ground between panel studies, which track the same individuals over time, and simple cross-sectional studies that observe individuals at one point in time. The approach we use here takes independent samples of individuals from population cohorts at multiple time points. This approach draws on the work of Borjas [15,16] who developed it to examine earnings trajectories by nativity. There are parallels with age-period-cohort analyses in that it estimates duration of residence effects for immigrants, period effects with survey year, and arrival cohort effects; however, our analyses also control for age. The inclusion of U.S.-born individuals enables U.S. to estimate the age and period effects from their sample and avoid complete multicollinearity among these time-dependent variables. This approach has been used in other studies examining immigrant-native health differentials [10,17,18].

We conduct analyses separately for men and women based on the previously noted evidence that these processes differ by gender [8,19]. We contrast the findings from conventional regression analyses that simply include period and duration of residence effects, to two alternative methods of assessing arrival cohort effects: the first accounts for baseline differences in smoking among arrival cohorts, while the second examines smoking probabilities by tracking foreign-born arrival cohorts as they increase their duration of U.S. residence. Our findings are thus a more rigorous test of the nativity difference in smoking than has previously been conducted. Considering that immigrant smoking patterns have implications for the Latino immigrant mortality advantage in coming decades [20], our study can provide insight into whether the role of smoking in the Latino immigrant mortality advantage can be expected to wane or persist in the future.

## 2. Materials and Methods

### 2.1. Data and Sample

We analyzed data from the U.S. National Health Interview Survey, a nationally representative health survey of the non-institutionalized population conducted by the U.S. Census Bureau using personal household interviews. Data were acquired through the Integrated Health Interview Series [21]. In order to track cohorts as they increase their U.S. duration of stay, we used survey waves spaced 5 years apart (1998, 2003, 2008, and 2013), which allowed us to follow the 1998 arrival cohort through to 2008, the 2003 arrival cohort through 2013, and the 2008 cohort through 2013. We were unable to follow the 1998 cohort through 2013 because the years in U.S. variable is censored at 15+ years, which makes this group heterogeneous in both arrival cohort and other characteristics. We limited the analytic sample to U.S.-born and foreign-born Latinos (Puerto Ricans born outside the U.S. are considered foreign-born, as they more closely resemble foreign-born Latinos), ages 20–64. We also excluded foreign-born Latinos who have lived in the U.S. 15 or more years because we could not distinguish their arrival cohort due to the censoring of this variable. Thus, our analytic sample included U.S.-born Latinos and Latino immigrants who arrived in the U.S. between 1984 and 2013. Of the 12,839 respondents meeting inclusion criteria, 347 cases (less than 3% of the sample) were excluded through listwise deletion due to missing data. This was well below the 5% threshold at which imputation is recommended [22]. The final total analytic sample size was n = 12,492. This study uses publicly available anonymous data and thus is exempt from human subjects review.

### 2.2. Measures

#### 2.2.1. Dependent Variable

*Current smoking status* (coded as current vs. former or never) was determined using two questions: “Have you ever smoked 100 cigarettes in your life?” and “How often do you smoke now—every day, some days, not at all.” Respondents were coded as current smokers if they have ever smoked 100 cigarettes in their lifetime and currently smoke every day or some days, and coded as former/never smokers if they do not currently smoke or have not smoked at least 100 cigarettes in their lifetime.

#### 2.2.2. Key Independent Variables

*Nativity*/*duration of U.S. stay* was coded as: U.S.-born (reference), foreign-born (FB) < 5 years in U.S., 5–9 years in U.S., 10–14 years in U.S. *Survey year* included 1998 (reference), 2003, 2008, and 2013. We created an *immigrant arrival cohort* variable by subtracting the duration of U.S. residence from the survey year (2004–2013 is the reference group; 1999–2003; and 1984–1998).

#### 2.2.3. Control Variables

*Age* (20–64) was measured continuously, and a quadratic term for age was included based on previous studies finding a curvilinear association of age and smoking [23]. We additionally control for education (less than primary, primary, secondary, and college or higher), and *language of interview* (English or Spanish) [24].

### 2.3. Statistical Analyses

Analyses were conducted in Stata 13, weighted to account for the NHIS multi-stage complex survey design. For our pooled cross-sectional analyses, we adjusted person weights to account for combining waves. Because of the large gender differences in smoking, all analyses were stratified by gender, consistent with previous tobacco studies [8].

We compared results from several alternative estimations to demonstrate the different conclusions drawn about the nativity differential in smoking. The first analysis used the conventional cross-sectional method, which included period and duration of residence as covariates, and we compared this to two alternative approaches that assessed the impact of immigrant arrival cohort. The first cohort approach examined baseline differences in smoking among arrival cohorts, while the second tracked foreign-born arrival cohorts as they increased their duration of U.S. residence. To summarize, we conducted the following three analyses: (1) a conventional pooled cross-sectional analysis adjusting for period and duration; (2) a regression accounting for arrival cohort; and (3) a repeated cross-section longitudinal cohort method to track cohorts.

The first analysis took the conventional cross-sectional approach, which examined differences in smoking between the U.S.-born and immigrants of differing duration of U.S. residence, and trends in smoking over time. To do this, we pooled across all survey waves and estimated a binomial logistic regression that assessed the association between duration and smoking, controlling for survey year and covariates. This approach examined period and duration differences, but masked the impact of immigrant arrival cohorts.

Our second analysis re-estimated the duration effect, this time accounting for arrival cohort by adding a term to the logistic regression for immigrant arrival cohort. The cohort term assessed whether immigrant cohorts differed in their smoking upon arrival in the U.S. (with the comparison group being the 2004–2013 arrival cohort). In this regression, the duration term can be understood as the association between duration of U.S. residence and smoking, while accounting for (baseline) differences in smoking upon immigrants’ arrival in the U.S.

The final analysis examined how U.S.-born and immigrants’ smoking prevalence differed over the period 1998–2013, additionally estimating whether different arrival cohorts displayed unique smoking trends relative to the U.S.-born over time in the U.S. To do this, we estimated a logistic regression with an interaction term between duration of U.S. residence and period (survey year). From the fitted equations, we calculated the predicted probabilities for current smoking at each time point for the U.S.-born, and for the foreign-born with 0–4 years and 5–9 years of U.S. residence. We followed three immigrant arrival cohorts (FB < 5 years. in 1998, 2003 and 2008). The 1994–1998 and 1999–2003 cohorts were followed for 10 years, through 2008 and 2013, respectively, when they had achieved 10–14 years duration of U.S. residence, while the 2004–2008 cohort was only followed for 5 years, when they achieved 5–9 years in the US. The coding of immigrant arrival cohorts for this analysis was not equivalent to the coding of arrival cohort in the previous analysis because of the spacing of the survey waves; therefore results about arrival cohort should be interpreted distinctly for these two analyses. We tested for differences between predicted probabilities using adjusted Wald tests (reporting F-statistics), with Šidák-Holm correction for multiple comparisons. Due to very low smoking prevalence among Latino immigrant women, we only used this repeated longitudinal cohort method among men.

## 3. Results

Table 1 presents the weighted socio-demographic characteristics of U.S. Latinos by gender from the pooled data spanning 1998–2013. The average age was 36 for men and 38 for women. A majority of U.S. Latinos (52%) completed secondary education, with females having slightly higher rates of college education than men (14% vs. 12%). Two-thirds completed the survey interview in English. Among men, the U.S.-born made up 58% of Latinos in our sample, compared to 62% of women. Immigrants who had been in the U.S. between 10 and 14 years at the time of interview made up approximately 15% of Latinos, followed by approximately 14% who had lived in the U.S. 5–9 years at interview. Among immigrants, most arrived prior to 1998 (18.4%), 15.5% arrived between 1999 and 2003, and 8.1% arrived between 2004 and 2013. Current smoking was more common among men (23%) compared to women (11%).

To examine the association of duration of residence with current smoking, we conducted regressions using three alternative specification methods, as described above. Table 2 provides the results for the first two approaches. For the conventional cross-sectional approach (Model 1), which examined the association between duration and smoking controlling for survey wave, we found that being foreign-born was associated with lower odds of current smoking relative to the U.S.-born, for both men and women. For example, immigrant men who had lived in the U.S. for less than five years had 0.72 times the odds of smoking compared to U.S.-born Latinos (95% CI 0.52, 0.99), and those with 10–14 years in the U.S. had 0.46 times the odds compared to U.S.-born Latinos (95% CI 0.35, 0.61). Moreover, all three categories of immigrants by duration of residence differed significantly from each other in odds ratios of current smoking (*F* = 3.76, *p* < 0.05). Among women, the odds of current smoking were also lower among the foreign-born compared with the U.S.-born. Recently arrived immigrant women had 0.52 times the odds of current smoking relative to their U.S.-born counterparts (95% CI 0.35, 0.77), immigrant women with 5–9 years had 0.35 times the odds (95% CI 0.22, 0.56) and those who had lived in the U.S. for 10–14 years had even lower odds relative to the U.S.-born (*OR* = 0.42, 95% CI 0.30, 0.58). Unlike for men, however, among immigrant women, the duration of residence groups do not differ significantly from each other in odds of current smoking (*F* = 1.35, *ns*).

The second specification added immigrant arrival cohort to the model (Table 2, Model 2). The arrival cohort terms controlled for differences in smoking prevalence at entry among immigrant cohorts entering in 1984–1998, 1999–2003, or 2004–2013 (reference). Under this specification, the association between arrival cohort and smoking was not statistically significant, for men or women, meaning that the separate arrival cohorts did not statistically differ in their smoking prevalence at entry into the U.S. The association between duration of U.S. residence and smoking was similar for men after controlling for arrival cohort, although only the 10–14 year immigrant category remained statistically different from their U.S.-born counterparts (*OR* = 0.50, 95% CI 0.31, 0.82); the overall effect of duration of U.S. residence was no longer statistically significant (*F* = 2.20, *p* = 0.11). For women, the association between duration of residence and smoking remained statistically significant and even strengthened slightly when accounting for arrival cohorts, with slightly lower odds ratios among all immigrant duration of residence groups compared with the odds ratios from the conventional analysis. We tested for multicollinearity in Models 1 and 2, and for both models, all VIFs remained below 10.

Table 3 presents the final approach, which examined tracked arrival cohorts and U.S.-born Latinos over time. We conducted this analysis on men only because the cell sizes for women who smoked among each arrival cohort/duration of residence combination precluded such an analysis. The predicted probabilities of smoking for each immigrant arrival cohort and the U.S.-born at each time point revealed some differences by arrival cohort, although the 95% confidence intervals were large, likely due to the small cell sizes when examining the combination of duration of residence and arrival cohort.

**Figure 1:**
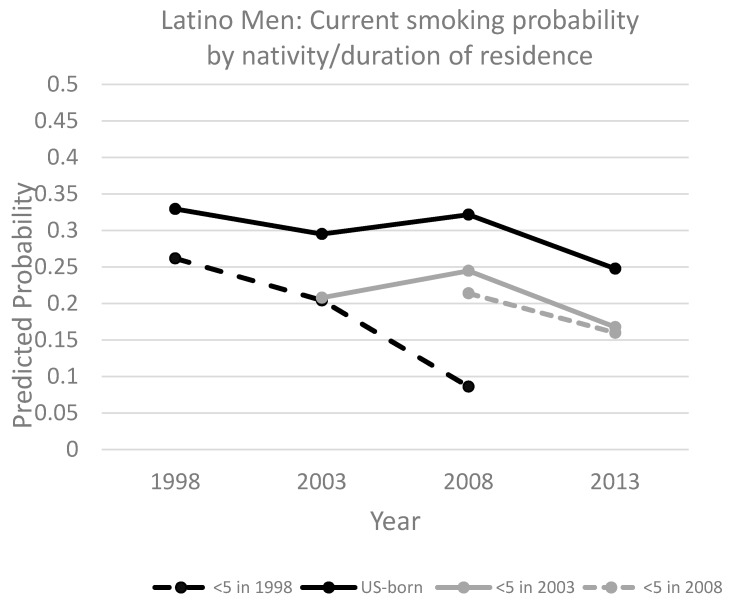
Predicted prevalence of current smoking among Latino men, comparing U.S.-born and immigrants by arrival cohort (cohort tracking method). Data come from U.S. NHIS, 1998–2013 [21].

Figure 1 plots the predictions for Latino immigrant men to better visualize the smoking trend over the period for the U.S.-born and each immigrant arrival cohort. All groups experienced an overall decline in smoking over time (Figure 1). However, the trends in smoking prevalence differed for U.S.-born Latinos compared with certain immigrant cohorts. Among the U.S.-born, there was little change in smoking prevalence during the 1998–2013 period, with the only significant difference being between a predicted prevalence of 32.9% in 1998 and 24.8% in 2013 (*F* = 8.47, *p* < 0.01). Among immigrants, the 1998 arrival cohort (entered the U.S. in 1994–1998) experienced a sharp decline in smoking prevalence between 1998 and 2008 (the furthest time point we can track them), from 26.2% to 8.6% (*F* = 8.18, *p* < 0.01). For the 2003 arrival cohort, smoking increased slightly, to 24.5%, between 2003 and 2008, and then declined to 16.8% through 2013; however, these differences were not statistically significant (*F* = 1.12, *ns*). Overall, the decrease among the 2003 arrival cohort (between 2003 and 2013) was less than 5% compared to over 15% for the 1998 arrival cohort (between 1998 and 2008). Finally, the 2008 arrival cohort experienced a decline, from 21.4% in 2008 to 16% in 2013, but this difference was not statistically significant (*F* = 1.01, *ns*).

## 4. Discussion

This study aimed to fill gaps in scholars’ understanding of nativity disparities in smoking by examining the roles of period, duration of U.S. residence, and immigrant arrival cohort in patterning smoking prevalence. Overall, findings confirm and extend existing research on the nativity difference in smoking, and how it has changed over time. We find that immigrants have maintained lower prevalence of current smoking compared with U.S.-born Latinos over the period 1998–2013, and that longer duration of U.S. residence is associated with lower odds of smoking among men. Going beyond existing research, we documented the impact of arrival cohort, providing two novel findings specific to Latino immigrant men: (1) accounting for immigrant arrival cohort slightly decreases the magnitude of the protective effect of duration of residence; and (2) the earliest arrival cohort of Latino immigrant men experienced the steepest decline in smoking over duration of U.S. residence. These findings, particularly those from the cohort tracking analysis, are suggestive of the importance of immigrant arrival cohorts in contributing to duration of residence differences in smoking, especially for men. Below, we discuss these findings in more detail, as well as their methodological and theoretical implications for studies of immigrant smoking and the Hispanic paradox.

A major methodological contribution of this study is the comparison of results from three alternative estimations, which revealed the different evidence about the nativity difference in smoking. The conventional approach showed a strong protective effect of duration of residence for men. The second approach, which added arrival cohort terms to the regression model, showed that for men, the effect of duration was slightly tempered when accounting for baseline differences in smoking among cohorts. We acknowledge, however, that the difference in the duration coefficients after accounting for arrival cohort was small and we cannot rule out the possibility that loss of statistical significance may have been due to multicollinearity. However, our final cohort tracking method paints a more distinct picture about the impact of arrival cohort on smoking. This method used smaller year intervals for arrival cohort and suggests that not all immigrant cohorts experience the same changes in smoking prevalence with duration of U.S. residence.

We found lower smoking among Latino immigrants than U.S.-born Latinos among both women and men, which is consistent with previous research [3]. Our results showed that this advantage remained fairly constant over the period 1998–2013, with immigrants’ smoking prevalence being lower than U.S.-born Latinos at each time point. Results also showed that duration of U.S. residence has protective effects against smoking among men, with lower odds of smoking among immigrant men of longer duration of residence. Taken together with results from a study finding lower smoking with greater acculturation among men [25], these findings add nuance to our understanding of the gendered way in which health behaviors change with time in the U.S. and acculturation.

By contrast, we found no difference in smoking across duration of residence categories for women. This finding is consistent with some previous research utilizing a nationally representative dataset (the 2002–2003 National Latino and Asian American Study) [8]. While other studies have shown increased smoking with acculturation (including duration of residence) among women [26], they have tended to be qualitative studies in smaller samples and from smaller geographic areas (e.g., San Francisco or Los Angeles, California), which makes them difficult to compare to our nationally representative sample of Latinos.

Our data also suggest that the earliest arrival cohort (entering 1994–1998) stands out because it experienced a sharper decline in smoking than the concurrent decline among the U.S.-born. There are several potential explanations for the steeper decline in smoking among this arrival cohort, which had the highest smoking rate upon arrival. First, it may be due to the fact that this early cohort arrived in the U.S. during a period of high Latin American immigration (which subsequently declined after the Great Recession) [27,28]; being part of a high migration cohort may mean those immigrants are less selective on smoking than subsequent arrivals. Consistent with the notion that the earlier arrival cohort may have been less selective on smoking, one study found no difference in odds of smoking between Mexican immigrants in the U.S. and non-migrants in Mexico during the period 1997–2007, which suggests immigrants from that period were not positively selected on smoking [29]. Being a less selective immigrant cohort (with higher smoking) may, in turn, make them more likely to quit smoking with time in the US.

Second, this early arrival cohort was not exposed to a restrictive tobacco environment in their sending countries and was thus potentially more sensitive to the rapidly increasing tobacco control policies in the U.S. during this period. For example, Mexican immigrants in this cohort left the country before tobacco control policies were implemented country-wide in the 2000s [30]. The tobacco environment in immigrants’ origin countries could not only explain their higher smoking at arrival in the US, but could also underpin their steep decline after they moved to the US. Immigrants in more recent cohorts may have already been exposed to tobacco control efforts before migrating and were less affected by the U.S. tobacco policies.

An alternative explanation could be that selective return migration from the U.S. to immigrants’ origin countries affected the smoking prevalence of the immigrants remaining in the US. There are few studies that provide evidence about the magnitude and nature of selective return migration of Latino immigrants to their origin countries, and most such existing studies are specific to Mexican immigrants. For example, one study found higher smoking rates among return migrants compared to non-migrants in Mexico [31]. However, in order for return migration to bias our findings about arrival cohorts, there would have to be evidence that return migration is more likely among certain arrival cohorts (that is, those who returned to their origin country were disproportionately from a particular arrival cohort); there is limited evidence to support this. Further, other studies have found return migration does not substantially bias their estimates for duration of residence differences in smoking [29]. Considering the evidence from prior studies finding little return migration bias and the lack of evidence about return migration being disproportionate across arrival cohorts, we contend that these results do not merely reflect the impact of return migration.

These results should be interpreted with several limitations in mind. Because of the low smoking among women, we were unable to track foreign-born women’s smoking probabilities, therefore results from the cohort tracking analysis speak only to foreign-born Latino men. Several data limitations should be considered when interpreting these findings. First, due to the complex sample design, we were unable to test the change in coefficients across models using likelihood ratio tests [32]. Additionally, due to the censoring of the duration of U.S. stay variable in the NHIS, we could only track cohorts through 14 years in U.S. before they were lost in the heterogeneous category of 15 or more years in the US. The results from the cohort tracking analysis should be interpreted with caution considering the large confidence intervals, but they are nevertheless suggestive of the importance of arrival cohort. This study provides novel information about the potential bias from arrival cohorts in traditional studies that pool multiple years of data to study immigrant smoking. Our repeated cross-sectional data permit the parsing out of the effects of duration in the U.S. from the effect of newly arrived immigrant cohorts, but are limited in the longitudinal assessment of duration in the U.S. because they do not track the same individuals. For example, if a substantial number of immigrants from a particular cohort left the US, this could bias the effects of duration in the US. The ideal data to address our research question would be multiple cohort studies that track the same individuals, from several new immigrant arrival cohorts, over time. However, given the lack of such data, our study uses nationally representative data and a research design that appropriately distinguishes arrival cohort effects—an important contribution to a literature that has largely relied on single time-point cross-sectional data to examine time in the U.S. effects on smoking, and often ignored the influence of entry cohorts. Finally, although not a focus of this study, future research should consider additional factors known to be important in understanding nativity differences in smoking. First, some research suggests that an earlier age at migration is a risk factor for smoking for immigrant women, although the relationship is less clear for men [33]. Second, smoking intensity in addition to prevalence, considering the nativity differences in the intensity of smoking (e.g., number of cigarettes per day), also has implications for health.

These findings have methodological and theoretical implications for interventions, policy, and future research. Importantly, our results are suggestive that cohort effects may be a non-trivial source of bias in cross-sectional studies that examine the relationship between duration of residence and health; future studies should use methods that assess arrival cohort effects. Considering the changing tobacco contexts in immigrants’ origin countries, smoking prevention and cessation interventions should take into account not only time in the US, but also the period during which immigrant cohorts arrived in the US, as well as the smoking trends in immigrants’ origin countries [34,35,36], particularly at the time the immigrant cohort left. For instance, if an immigrant arrives in the U.S. at a time when smoking is already low, the decrease in smoking may be lower in magnitude. Furthermore, findings speak to the importance of global tobacco control surveillance and policy. Monitoring changes in tobacco prevalence in Latin American immigrant sending countries might improve our ability to anticipate smoking trends among newly arrived cohorts of immigrants [36]. Finally, studies of immigrant health should consider how immigrant arrival cohorts vary based on dynamic origin country contexts.

## 5. Conclusions

In the beginning of the paper, we noted that understanding the persistence of the smoking differential between immigrant and U.S.-born Latinos could offer insight into the future persistence of the Latino mortality paradox. Overall, this study presented evidence that lower smoking prevalence among immigrants compared to U.S.-born Latinos, which appears to be a key reason for their mortality advantage according to prior studies, persists even in the context of declining smoking in immigrant sending countries. However, prior immigrant arrival cohorts may have had an even greater advantage than immigrants who have arrived since 2003 because of the sharp decline in smoking during their early years of stay in the US. If the recent patterns continue, Latino immigrants may retain their mortality advantage over U.S.-born Latinos for some time, but the fact that the earliest cohort experienced the greater smoking decline may be a sign that, in the context of declining smoking in the U.S. and other countries, immigrants’ advantage may not be as large as in the past. Thus, our findings beg an important question: will Latino immigrants’ low smoking remain as salient a factor in shaping the Latino mortality advantage in the future?

## Figures and Tables

**Table 1:** Weighted sample characteristics by gender: U.S. Latinos, 1998–2013.

	Men	Women
	% or Mean	% or Mean
Age (mean)	36.3	38.1
Education		
Less than primary	6.1	5.6
Primary	29.3	27.5
Secondary	52.4	52.7
College	12.2	14.2
Language of interview		
English	66.6	67.3
Spanish	33.4	32.7
Survey year		
1998	18.3	17.4
2003	23.6	24.4
2008	28.0	27.1
2013	30.0	31.1
Nativity/Duration of U.S. residence		
U.S.-born Latino	57.9	62.0
<5 years in U.S.	11.3	8.5
5–9 years in U.S.	14.5	14.3
10–14 years in U.S.	16.3	15.1
Immigrant arrival cohort		
Prior to 1998	18.4	17.6
1999–2003	15.5	13.2
2004 or later	8.1	7.2
Current smoker	23.3	11.3

Note: Data come from U.S. NHIS, 1998–2013 [21].

**Table 2:** Logistic regressions predicting current smoking among Latinos by gender: Cross-sectional analyses.

	Model 1 Conventional Analysis		Model 2 Arrival Cohort Analysis	
	Men	Women	Men	Women
	AOR (95% CI)	AOR (95% CI)	AOR (95% CI)	AOR (95% CI)
Duration of U.S. stay (U.S.-born = reference)				
<5 years	0.72 *	0.52 **	0.72	0.46 **
	(0.52–0.99)	(0.35–0.77)	(0.48–1.08)	(0.26–0.83)
5–9 years	0.66 **	0.35 ***	0.69 +	0.29 **
	(0.49–0.89)	(0.22–0.56)	(0.45–1.05)	(0.12–0.66)
10–14 years	0.46 ***	0.42 ***	0.50 **	0.32 **
	(0.35–0.61)	(0.30–0.58)	(0.31–0.82)	(0.15–0.68)
Survey year (1998 = ref)				
2003	0.79 *	0.84	0.77 *	0.85
	(0.64–0.97)	(0.68–1.04)	(0.63–0.95)	(0.69–1.05)
2008	0.87	0.88	0.84	0.91
	(0.68–1.13)	(0.69–1.11)	(0.64–1.08)	(0.71–1.15)
2013	0.74 **	0.55 ***	0.70 **	0.58 ***
	(0.59–0.93)	(0.44–0.69)	(0.54–0.90)	(0.46–0.73)
Arrival cohort (2004 or later = ref)				
Entered in 1998 or earlier	--	--	0.87	1.39
			(0.55–1.38)	(0.68–2.85)
Entered in 1999–2003	--	--	1.04	1.12
			(0.69–1.57)	(0.54–2.31)
N	5313	6970	5313	6970

Note: Data come from U.S. NHIS, 1998–2013 [21]. Model includes controls for age, age-squared, education, and language of interview. Standard errors in parentheses, *** *p* < 0.001, ** *p* < 0.01, * *p* < 0.05, + *p* < 0.10.

**Table 3:** Predicted prevalence of smoking among Latino men by immigrant cohort: Tracking method.

Immigrants by Arrival Cohort	Margin	95% CI	
Entered in 1998			
<5 in 1998	26.2	14.9	37.5
5–9 in 2003	20.4	12.8	28.1
10–14 in 2008	8.6	4.0	13.3
Entered in 2003			
<5 in 03	20.8	14.8	26.8
5–9 in 2008	24.5	15.2	33.7
10–14 in 2013	16.8	11.0	22.5
Entered in 2008			
<5 in 2008	21.4	12.8	30.0
5–9 in 2013	16.0	9.2	22.8
U.S.-born			
1998	32.9	28.4	37.5
2003	29.5	25.0	34.0
2008	32.2	26.2	38.1
2013	24.8	20.8	28.7

Note: Predicted probabilities calculated from a regression (see Table S1) that includes an interaction between survey year and duration of U.S. residence. Data come from U.S. NHIS, 1998–2013 [21].

## Supplementary Materials

The following are available online at www.mdpi.com/1660-4601/14/3/255/s1, Table S1: Logistic regressions predicting current smoking among Hispanic men (period and duration interaction).
