# Supplementary material for: Smoking Trends among U.S. Latinos, 1998–2013: The Impact of Immigrant Arrival Cohort
Source: Int J Environ Res Public Health. 2017 Mar 2;14(3):255. doi: 10.3390/ijerph14030255 (PMC5369091; doi:10.3390/ijerph14030255)
Supplement: Supplementary file 1 [file ijerph-14-00255-s001.pdf]

Supplementary Materials: Smoking Trends among U.S. Latinos, 1998–2013: The Impact of Immigrant Arrival Cohort

Georgiana Bostean*, Annie Ro and Nancy L. Fleischer

Table S1. Logistic regressions predicting current smoking among Hispanic men (period and duration interaction).

**Survey Year (1998 = Ref)**	
2003	0.84
	(0.11)
2008	1.00
	(0.16)
2013	0.71 *
	(0.10)
Duration of U.S. stay (US-born = ref)	
<5 years	0.72
	(0.23)
5–9 years	0.72
	(0.16)
10–14 years	0.61 *
	(0.14)
Period * duration interaction	
2003 * US-born (reference)	1.00
	(0.00)
2003 * < 5 years	0.87
	(0.32)
2003 * 5–9 years	0.85
	(0.27)
2003 * 10–14 years	0.86
	(0.26)
2008 * US-born (reference)	1.00
	(0.00)
2008 * < 5 years	0.79
	(0.33)
2008 * 5–9 years	0.95
	(0.34)
2008 * 10–14 years	0.33 **
	(0.13)
2013 * US-born (reference)	1.00
	(0.00)
2013 * < 5 years	2.04
	(1.03)
2013 * 5–9 years	0.81
	(0.28)
2013 * 10–14 years	1.01
	(0.31)
n	5313

Notes: Model controls for age, age-squared, education, and language of interview; standard errors in parentheses; *** $p < 0.001$, ** $p < 0.01$, * $p < 0.05$, + $p < 0.10$.

© 2017 by the authors. Submitted for possible open access publication under the terms and conditions of the Creative Commons Attribution (CC-BY) license (http://creativecommons.org/licenses/by/4.0/).
